# Successful Removal of over Eleven Feet of Small Intestine for Mesenteric Thrombosis

**Published:** 1913-03

**Authors:** 


					﻿•m-
c
©
X
Ch F-
71	7
—
’■‘35 c
•_	?	o
o	“	XJ
k	Jg	o
od	.	.	<£
g	.2	u .
C	• X	.	£	>J -UP
K	5	r2	£	'*“'	"©	C ©
c	M	g	8	.£	8	F	<2 -«
g	g	-	c	fa	fa	q	fa
S’-1	oo	-	—	£	V	a.
o>-	51	2	~	fl m	00 ' — &€
2-	a	?	’	F fa	c S fa	§
a 5	§	-8	w	^	•" g .fa fa
Z	§	®	?	2	so	_r	, • fa	cS	fl o	§ >
2	L	.2	~	2	t	-g	o 'g	5	J	v	®
s «o	».. 5 -u +- o -a	c, fa p
So	o	.	•	’ii	—	p	.	P	c _	cp	i.	_,
x	Z	. fl p ~ S F w S a c . — ci .	.2c
o	u	g 2 2 5 S	o o F 2 fa	dpgc.Ca05
g	s	§s«^j.s	I’l’dl	FStlial
2	«H	8	F-(	£1 OJ	ajs	rt	erf	C.H A	fl	&
v	H	r - ■_ 2	z	c	c^oeP-i	fS^f^pof
g	e	fl g o fa10	~	y £ §	flp^flfl-fac
Z	fl fa _, 03 m	a	®	fa fa fl P C	ci +- fa O fl «S fa
M	S> a = 2 3 ® § ”fa?FOPogfaP§Fno
j o	540	8	74 T3 2 T1	72 rj .8 o fl ci^ bio
9 o	S fa 8 fl> £ 2 2? ^*SS^fl£’c.SS*>S£S
<	g	71	-S r—fl fl
Sc	ixapaoBrt -co, fa>--ofl 2 t> » fl g fl gc
co R	rn O 02 HH > fa fa ■< s 02 <1 fa hh O 02^50202^
§ e
H	5s	O ,	O O	O	O	o
a	,rH A	4--	4—	4->	4^
M	©	q *£	a	ci c:	ci	4fa	ci	ci
^>.	~ »3 9>	Tj +-> T	4fa 71	4—	4—
fa-	ci -5 X	rfl rS rfl ±	©	rj	O	74
3	1	«S=	2131	S fl §	f	■§>	§
g - S fa	S S 02 S t> 02 «	02 S 02	02
- fl
a ci
fat	fa*» ***• fa*« F*3 Jx- <*• '**• fa*^ »**< L*	L* F*i
>	rG	ZX	C> S S
°	,	P	>kkk>>>>>>>>kk>>	>>>>£
t,	'S	OJ	OOOcOOOpooOOOCOc	o C o o g
2	®	O	WOOyOOoOyOOOiyOOy	O C S 9 P
c	OOOoi-CpOyfUOOoOOy OCc^y
J 8	Ph M CP l± fa fa fa M pej fa 34 PS P4 Ph M (^ fa fa fa fa £
>	F
2	C5	-S'®
k2	B fa 4	^OOiolOOOCOio<©®C<IC>aOCOre	K5 o Iffl ® <a
g	g	g y S	<NC3KHb-t-COCOc3eoCO<M(NT-lrH2	fa fa O S
”	CmO	‘-Oinic-tiThfafacCcoMcococoeococ*	«cc m M g
u	e£	£3 <i>
P	”	<K
x	H
co
a
°	fa
P
co	K
2	_ fa	S fa J_ _. 00 O	rH fl
®	'f 2 B '2 H ® -H F fa fH g 'c 2 fa ‘C
g	BrFS5-3^|3s-S®52^£	^fafaflfa
2	^a>o'o-«.gFSg§^g2Sfe^	25^.2^
O	0 VI 0
2	faCqMfalOCOt-COOiGrHOqcO’^lOCO	t- X CS> o rH
Z;	fa fa t-H fa ,-H r-, fa,Hfa<Nc3
				

